# Initial Capture Failure With Delayed Resolution in Atrial Leadless Pacemaker Implantation: A Case of ATTR Amyloidosis With Sinus Node Dysfunction

**DOI:** 10.1002/joa3.70288

**Published:** 2026-02-08

**Authors:** Yasuyuki Takada, Junichi Kamoshida, Muryo Terasawa, Kazuhiro Satomi, Yoshinao Yazaki

**Affiliations:** ^1^ Department of Cardiology Tokyo Medical University Tokyo Japan

## Abstract

ATTR amyloidosis patient with initial atrial capture failure showed delayed threshold improvement over time. Stable current of injury and impedance guided expectant management, avoiding unnecessary device repositioning while achieving successful outcomes.

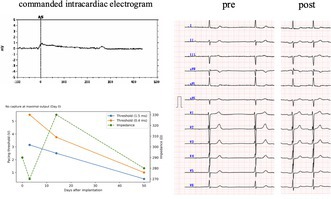

Transthyretin cardiac amyloidosis (ATTR) is frequently accompanied by conduction abnormalities, including sinus node dysfunction requiring permanent pacing. Dual‐chamber leadless pacemakers provide an option for patients with frailty, infection risk, or limited venous access [1]. During atrial leadless pacemaker implantation, however, elevated acute pacing thresholds or absent atrial capture create uncertainty regarding device stability and repositioning needs.

An 84‐year‐old woman with ATTR amyloidosis presented with recurrent syncope and exertional dyspnea. Cardiac imaging demonstrated diffuse left ventricular hypertrophy with apical sparing, and pyrophosphate scintigraphy showed increased myocardial uptake. Fat aspiration biopsy confirmed amyloid deposition. Ambulatory monitoring revealed profound bradycardia with recurrent asystolic pauses.

Considering the patient's diminished activities of daily living and the need to minimize postoperative mobility demands, we proceeded with implantation of a dual‐chamber leadless pacemaker (Aveir DR, Abbott). Following implantation of the right ventricular device, which exhibited favorable acute electrical parameters (threshold 0.75 V/0.4 ms; impedance 290 Ω), we proceeded with implantation of the atrial leadless pacemaker. The blood pool impedance measured in the inferior vena cava at that time was 260 Ω. Multiple pre‐mapping attempts were performed within the right atrial appendage; however, even at maximal output (6 V/1.5 ms), atrial capture was intermittent, and stable atrial capture could not be achieved. At a pulse width of 0.4 ms, no atrial capture was observed, even at maximal output. Despite the absence of capture, the current of injury (COI) characterized by ST‐segment elevation on the intracardiac electrogram, was clearly present (1 mV), and impedance remained stable (290 Ω; Figure 1).

**FIGURE 1:**
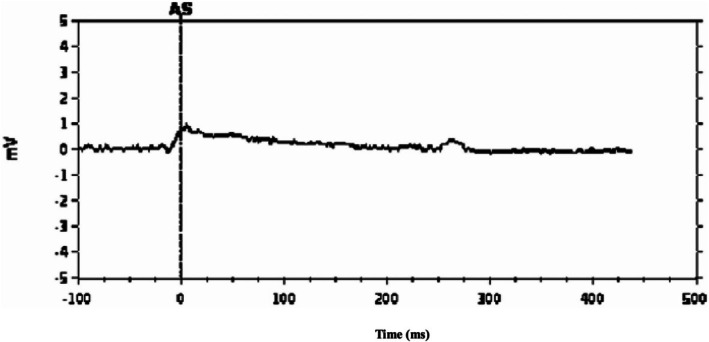
Commanded intracardiac electrogram during tether mode. Commanded intracardiac electrogram obtained during tether mode demonstrating a steep current of injury exceeding 1 mV in amplitude with a rapid upstroke. Despite the presence of current of injury and stable impedance (290 Ω), atrial capture could not be achieved at maximal output (6 V/1.5 ms).

We considered retrieving the atrial device and proceeding with right ventricular implantation alone. However, isolated right ventricular pacing in patients with sinus node dysfunction has been associated with an increased risk of heart failure [2]. In this patient, the underlying ATTR cardiomyopathy with diastolic dysfunction created conditions in which chronic right ventricular apical pacing would be expected to cause further hemodynamic deterioration. Therefore, following atrial leadless pacemaker implantation, we elected to observe whether the atrial pacing threshold would improve during the chronic phase. After a 30‐min observation period, the atrial pacing threshold remained unchanged; however, the COI and impedance were stable.

Previous reports in atrial leadless pacemaker implantation have shown that a steep increase COI ≧ 2 mV and impedance reflects adequate contact with viable atrial myocardium and is predictive of subsequent improvement in chronic pacing thresholds [3]. In our case, although the magnitude of COI elevation was limited to approximately 1 mV, the increase was steep, and an increase in local impedance relative to the blood pool impedance was observed at the implantation site.

In conventional transvenous pacemaker systems with active‐fixation leads, a sustained positive deflection of the current of injury measured at 80 milliseconds after its onset (CI80) has been shown to reflect secure helix engagement and to predict subsequent improvement in pacing thresholds over the chronic phase, spanning several weeks to months [4]. In our case, CI80 also demonstrated a sustained positive deflection.

Accordingly, the atrial leadless pacemaker was left in situ, with the expectation of potential improvement in chronic‐phase thresholds (Figure 2). By postoperative Day 3, atrial capture appeared (3.0 V/1.5 ms; 5.5 V/0.4 ms). At 2 weeks, the thresholds improved (2.5 V/1.5 ms; 3.0 V/0.4 ms) and the impedance increased to 330 Ω. At 2 months, the thresholds further improved (0.5 V/1.5 ms; 1.0 V/0.4 ms) with impedance stabilizing at 280 Ω (Figure 3). The device was programmed to AAI mode at 50 beats per minute with VVI backup at 40 beats per minute. The patient predominantly maintained sinus rhythm with pacing support during occasional bradycardic episodes (Figure 4). Throughout the 4‐month follow‐up period, she has remained asymptomatic without recurrence of syncope or dyspnea.

**FIGURE 2:**
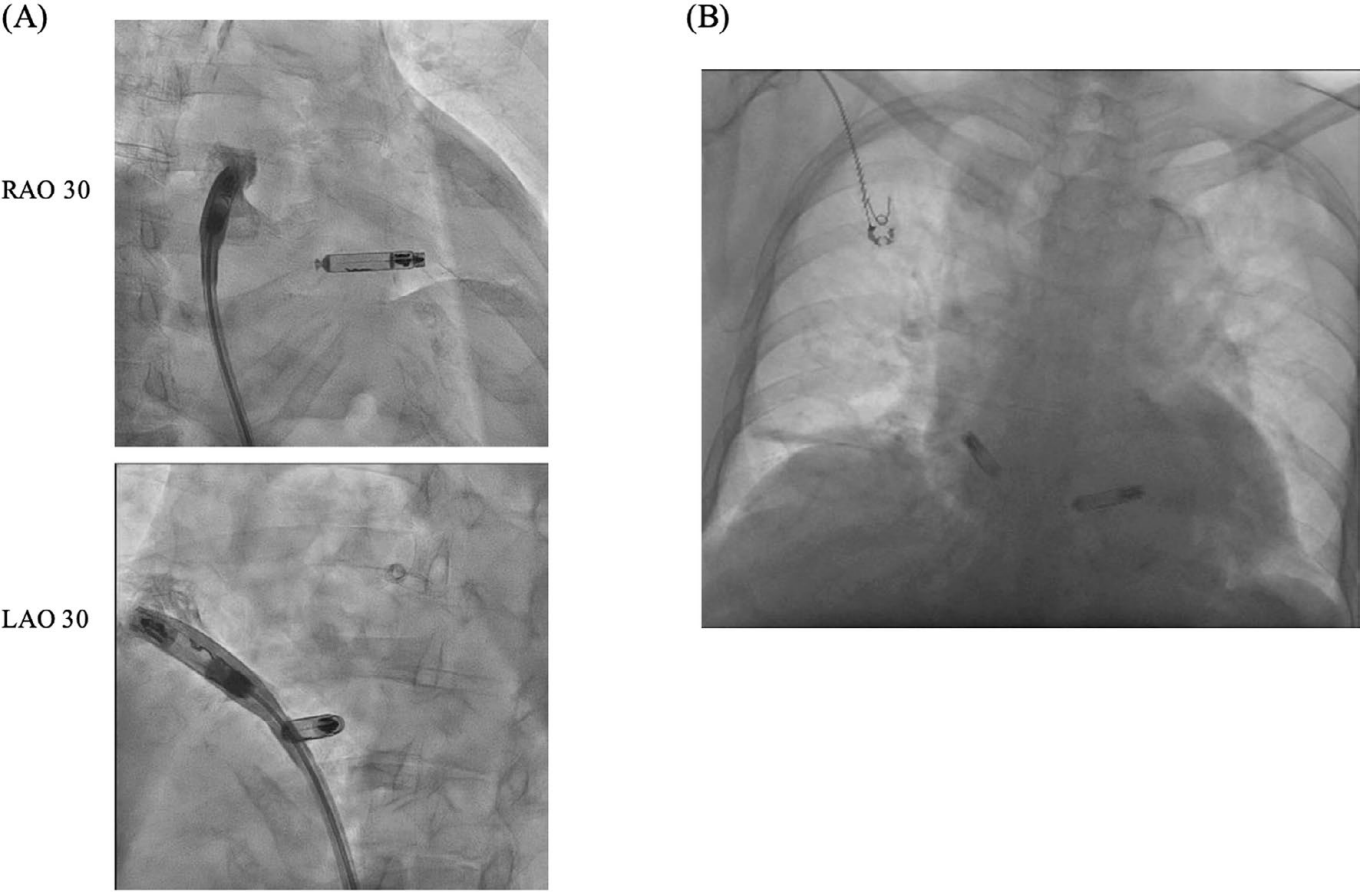
Fluoroscopic images during and after dual‐chamber leadless pacemaker implantation. (A) Fluoroscopic views during the procedure. Right anterior oblique (RAO) and left anterior oblique (LAO) projections showing the ventricular leadless pacemaker positioned in the right ventricle. Contrast injection through the atrial leadless pacemaker delivery catheter delineates the right atrial appendage. (B) Chest radiograph obtained immediately post‐implantation showing final positions of both atrial and ventricular leadless devices. The atrial device is positioned in the right atrial appendage and the ventricular device in the right ventricular.

**FIGURE 3:**
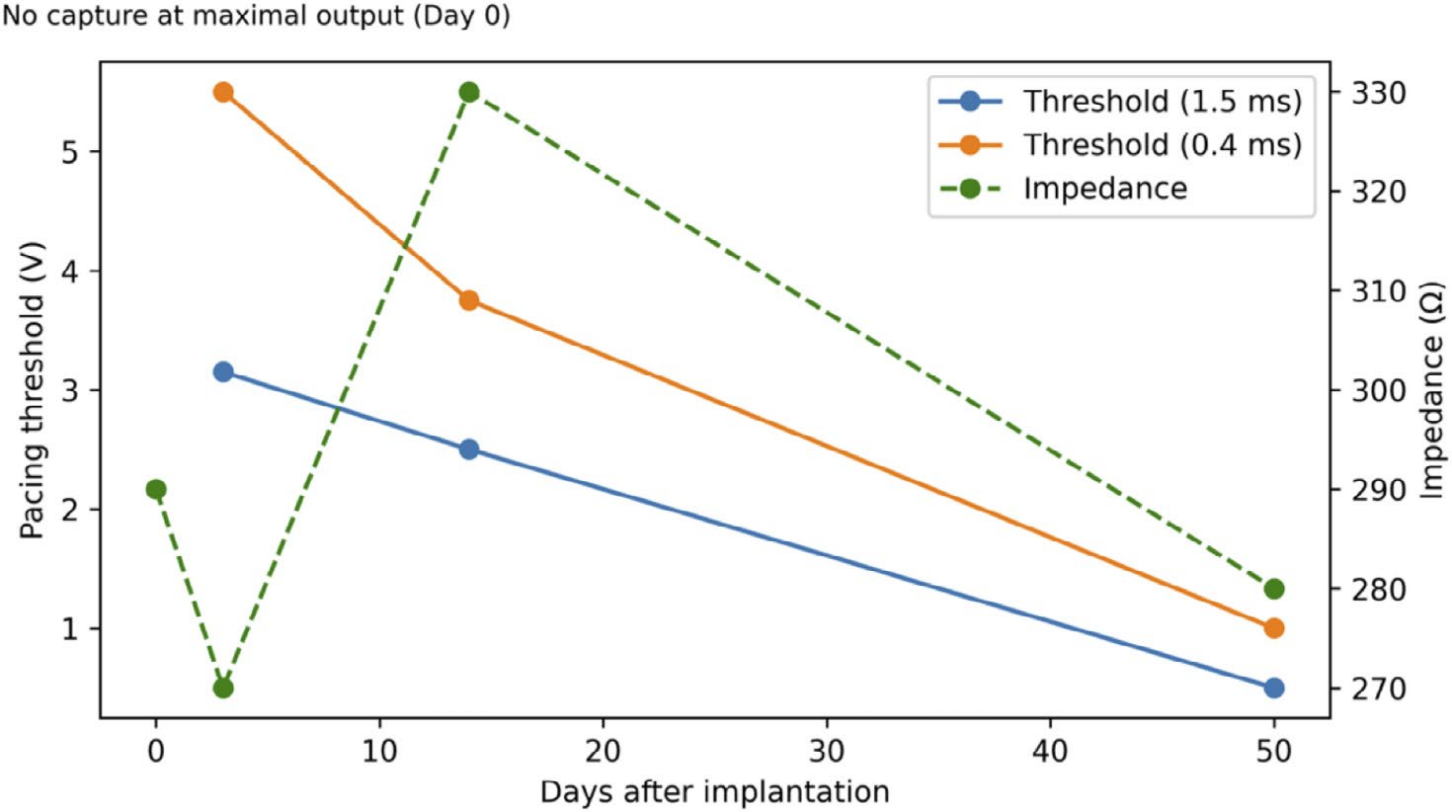
Time course of pacing thresholds and impedance following atrial leadless pacemaker implantation. Pacing capture thresholds at pulse widths of 0.4 and 1.5 ms and impedance over time following atrial leadless pacemaker implantation. Atrial capture first appeared on postoperative Day 3, with progressive improvement in thresholds observed at 2 weeks and 2 months.

**FIGURE 4:**
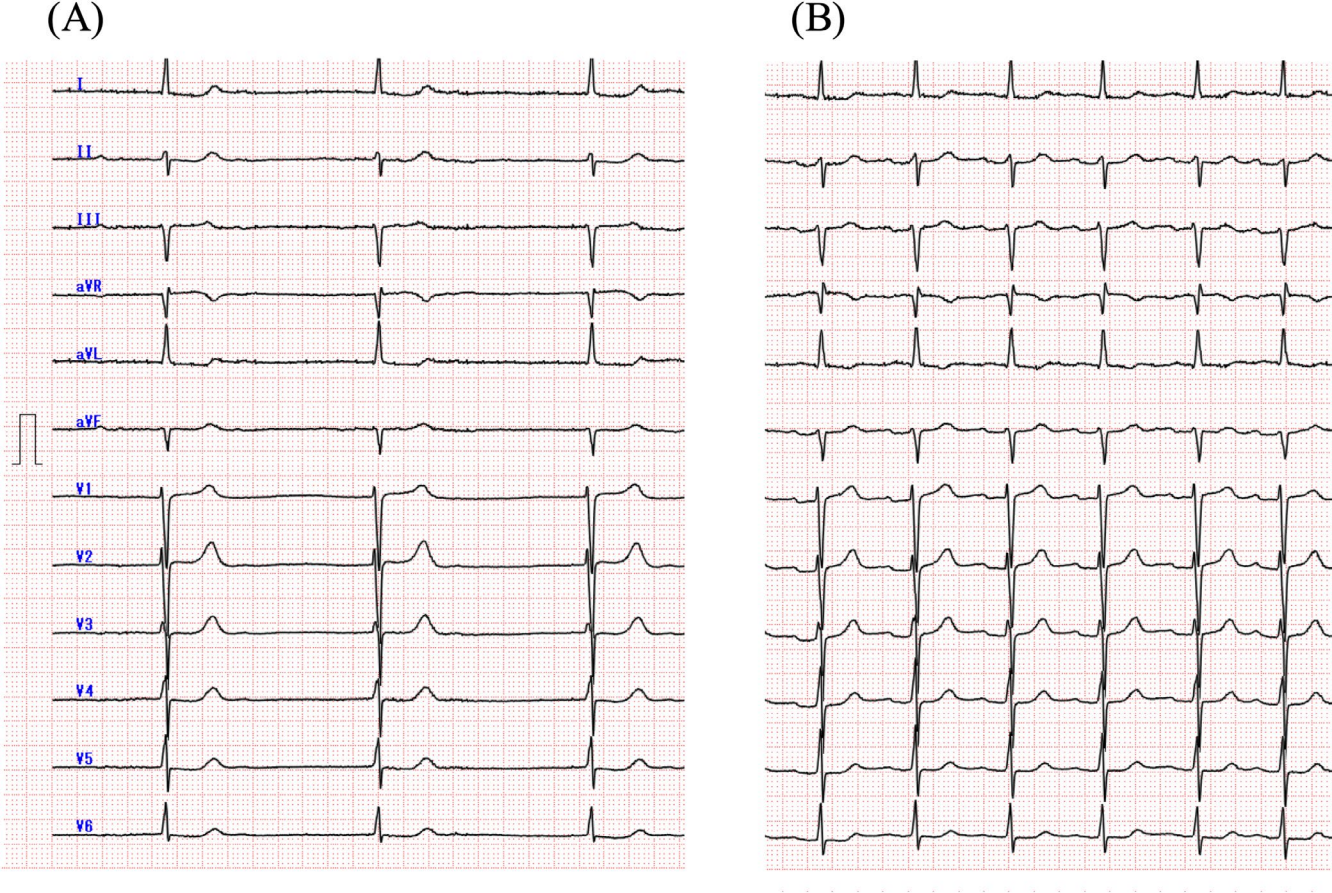
12‐lead electrocardiograms before and after dual‐chamber leadless pacemaker implantation. (A) Baseline electrocardiogram at presentation showing junctional rhythm at 31 beats per minute with absent P waves, representing one of the bradycardic episodes associated with sinus node dysfunction. (B) Post‐implantation electrocardiogram at 2 weeks demonstrating sinus rhythm at 62 beats per minute. The dual‐chamber leadless pacemaker occasionally provides backup pacing when bradycardia occurs.

This case suggests that even in the absence of acute atrial capture, clearly defined indices based on current of injury and impedance may help predict subsequent chronic improvement. Although acute threshold variations have been reported in leadless systems, this case provides specific insight into the threshold dynamics in ATTR amyloidosis, where initial capture failure may still result in favorable long‐term outcomes when guided by appropriate electrophysiological markers. In atrial leadless systems, acute threshold elevation is common and often improves within 24 h, with further decline over subsequent weeks. Avoiding unnecessary repositioning may reduce procedural risk without compromising long‐term performance. The right atrial appendage has complex and delicate anatomy. Repeated device manipulation in this region carries significant risk, including pericardial effusion and cardiac tamponade. Accurate intraoperative identification of adequate fixation is paramount to minimize unnecessary intervention. In our experience, a minimum observation period of 30 min with stable COI and impedance may be reasonable before considering device retrieval, though the optimal waiting time remains undefined. Criteria that might mandate device retrieval could include absence of COI, declining impedance, or inability to achieve any degree of capture despite repositioning attempts, although standardized guidelines for these decisions are needed. The mechanism underlying the initial absence of capture with subsequent chronic threshold improvement in this patient merits discussion. In cardiac amyloidosis, extracellular amyloid deposition at the electrode‐tissue interface can significantly elevate pacing thresholds. Previous case reports have documented dynamic threshold changes in AL amyloidosis, where threshold elevation correlated with disease activity and improved following systemic chemotherapy [5]. Our patient, however, had ATTR amyloidosis—a progressive condition without definitive treatment. The initial failure to capture likely resulted from acute inflammatory changes at the fixation site superimposed on underlying amyloid infiltration. The subsequent threshold decline over weeks suggests resolution of the acute inflammatory component, despite ongoing amyloid deposition. Whether this improvement will be sustained in the setting of progressive amyloid accumulation remains uncertain, emphasizing the importance of continued threshold surveillance. Development of additional electrophysiological markers predictive of long‐term device performance would strengthen the rationale for deferring acute repositioning in similar cases. Implantation of an atrial leadless pacemaker in patients with ATTR amyloidosis remains controversial, given the potential for elevated atrial pacing thresholds related to atrial myopathy and the additional health care–economic considerations associated with dual‐chamber leadless systems. Although our case suggests that the Aveir DR system can be feasible in carefully selected patients, evidence in this population remains limited. Therefore, further accumulation of similar cases and larger multicenter experience are essential to better define patient selection, expected atrial threshold behavior, and clinical and economic outcomes.

## Funding

The authors have nothing to report.

## Ethics Statement

The authors have nothing to report.

## Consent

Written informed consent for publication was obtained.

## Conflicts of Interest

The authors declare no conflicts of interest.

## Data Availability

The data that support the findings of this study are available on request from the corresponding author. The data are not publicly available due to privacy or ethical restrictions.
